# Sodium Houttuyniae attenuates ferroptosis by regulating TRAF6-c-Myc signaling pathways in lipopolysaccharide-induced acute lung injury (ALI)

**DOI:** 10.1186/s40360-024-00787-x

**Published:** 2024-09-06

**Authors:** Juan Li, Yan-ping Hu, Xing-ling Liang, Ming-wei Liu

**Affiliations:** 1 Department of Respiratory and Critical Care Medicine, Third People’s Hospital of Yuxi City, Yuxi, Yunnan 653100 China; 2 Department of Neurology, Third People’s Hospital of Yuxi City, Yuxi, Yunnan 653100 China; 3 Department of Emergency, The First Affiliated Hospital of Kunming Medical University, Kunming, Yunnan 650032 China; 4 Department of Emergency, People’s Hospital of Dali Bai Autonomous Prefecture, No. 35 Renmin South Road, Xiaguan Street, Dali, Yunnan 671000 China

**Keywords:** Sodium Houttuyniae, Acute lung injury, Ferroptosis, TRAF6-c-Myc pathway

## Abstract

The impact of Sodium Houttuyniae (SH) on lipopolysaccharide (LPS)-induced ALI has been investigated extensively. However, it remains ambiguous whether ferroptosis participates in this process. This study aimed to find out the impacts and probable mechanisms of SH on LPS-induced ferroptosis. A rat ALI model and type II alveolar epithelial (ATII) cell injury model were treated with LPS. Enzyme-linked immunosorbent assay (ELISA), hematoxylin-eosin (HE) staining, and Giemsa staining were executed to ascertain the effects of SH on LPS-induced ALI. Moreover, Transmission electron microscopy, Cell Counting Kit-8 (CCK8), ferrous iron colorimetric assay kit, Immunohistochemistry, Immunofluorescence, Reactive oxygen species assay kit, western blotting (Wb), and qRT-PCR examined the impacts of SH on LPS-induced ferroptosis and ferroptosis-related pathways. Theresults found that by using SH treatment, there was a remarkable attenuation of ALI by suppressing LPS-induced ferroptosis. Ferroptosis was demonstrated by a decline in the levels of glutathione peroxidase 4 (GPX4), FTH1, and glutathione (GSH) and a surge in the accumulation of malondialdehyde (MDA), reactive oxygen species (ROS), NOX1, NCOA4, and Fe^2+^, and disruption of mitochondrial structure, which were reversed by SH treatment. SH suppressed ferroptosis by regulating TRAF6-c-Myc in ALI rats and rat ATII cells. The results suggested that SH treatment attenuated LPS-induced ALI by repressing ferroptosis, and the mode of action can be linked to regulating the TRAF6-c-Myc signaling pathway in vivo and in vitro.

**Supplementary Information:**

The online version contains supplementary material available at 10.1186/s40360-024-00787-x.

## Introduction

Acute lung injury (ALI) stands out as a serious respiratory disease manifested by inflammation of the lungs and destruction of the lung tissue structure, with a lethality rate of 30–60% [1]. As a result, there is damage to both alveolar epithelial cells as well as capillary endothelial cells by different injury factors, leading to diffuse pulmonary interstitial, acute hypoxic respiratory insufficiency, and alveolar edema [2, 3]. Numerous pieces of literature have affirmed that the dominant causes of ALI are gram-negative bacterial infections, and lipopolysaccharide (LPS) stands out as the major component of the outer membrane of these bacteria, which can culminate inflammatory responses and lung injury [4, 5]. Therefore, LPS has been extensively used to establish experimental models for anti-ALI drug development, which can adequately trigger neutrophilic inflammatory responses and increase cytokine levels in the lungs. In addition, in an experimental ALI animal model, intratracheal LPS administration induces pulmonary inflammation and exacerbates lung tissue injury by enhancing reactive oxygen species (ROS) generation and activating inflammatory responses [6–9]. Therefore, inhibition of oxidative stress and inflammation has been the focus of research to develop novel therapeutic modalities for LPS-induced ALI.

Excessive pooling of lipid peroxides characterizes, ferroptosis, which denotes a form of cell death. Lipid ROS pileup is a hallmark of ferroptosis that minimizes the levels of GPX4 and glutathione (GSH), and as a consequence, cellular ferroptosisoccurs [10]. Recent studies have implied that ferroptosis takes part in the LPS-induced ALI process [11, 12]. For example, Bao et al. ascertained that liproxstatin-1 alleviated neutrophilic asthma and LPS/IL-13-triggered bronchial epithelial cell injury in mice by impeding ferroptosis [13], confirmed that ferrostatin-1 dampens acute lung injury caused by lipopolysaccharide via suppressing ferroptosis [9]. Inflammation and infection are the two known means that enhance iron chelation in cells. When there is an iron overload, oxidative stress, inflammatory responses, and dysfunction of the mitochondria occur. Uncontrolled free iron exerts harmful impacts on the tissues of the lung and culminates in damage to lung tissues through ferroptosis [14]. Hence, the suppression of ferroptosis can effectively dampen pathological destruction inflicted on lung tissues.

Houttuynia cordata Thunb is a perennial herbaceous plant that is edible and exhibits a discernible fishy smell that dominantly grows in moist and shady regions of East Asian countries for instance, Japan, China, and Korea [15, 16]. Houttuynin (decanoyl acetaldehyde, C12H22O2) is the main active ingredient of Houttuynia cordata Thunb and is unsteady owing to its rapid polymerization or oxidization. Sodium houttuyfonate (SH, C12H23NaO5S), an addition compound from sodium bisulfite and houttuynin, is more stable than houttuynin, and retain their main pharmacological activities, entailing antibacterial, antifungal, and anti-inflammatory activities [17]. Shen et al. affirmed that SH is an attractive candidate against idiopathic pulmonary fibrosis and bleomycin-induced pulmonary toxicity [18]. Yi et al. confirmed that SH relieves ventilator-induced lung injury by inhibiting the ROS-mediated JNK pathway [19]. Yang et al. ascertained that SH can effectively relieve the damage to alveolar structure and the generation of inflammatory factors in lung tissues throughout ALI, reduce the exudation of proteins in alveolar lavage and the degree of alveolar injury, and suppress not only the MAPK but also NF-κB signaling pathways activation [17]. Nonetheless, the protective impact of SH on LPS-induced ALI and the mechanisms involving it are still far from elucidated.

Given the above information, we investigated the impact of SH on LPS-induced ALI and further explored the possible molecular mechanisms in vivo and in vitro to avail an efficient and promising approach and theoretical basis for treating ALI.

## Materials and methods

### Drugs

Sodium Houttuyniae (Purity 99%) were acquired from Xi’an Shennong Biotechnology Co., Ltd (Xi’an, Shaanxi, China, Item No. SN-YXC2020510). Sodium Houttuyniae were identified and biochemical fingerprints obtained according to previously reported methods [20–22].

### Animal experiments

6–8 weeks old male Sprague Dawley (SD) rats (180–220 g body mass) were received from the Experimental Animal Center of Kunming Medical University (Kunming, China). The Ethics Committee of Kunming Medical University approved all animal experiments, and the study adhered to the guidelines of the Animal Care and Use Committee of the Kunming Medical University. Our study adheres to ARRIVE reporting guidelines.

36 rats were randomly divided into six groups (6 rats per group) as follows: a control group with intratracheal instillation of 1.5 mg/kg normal saline andan LPS group with similar site injection but using 5 mg/kg LPS; according to previous literature [23, 24], LPS + c25-140 (inhibitor of TRAF6, SparkJade, Shandong, China) group with intra-tracheal instillation of 5 mg/kg LPS accompanied by intraperitoneal injection of 20 µM c25-140; and LPS + SH group with intratracheal instillation of 5 mg/kg LPS accompanied by intraperitoneal injection of 10, 20, and 40 mg/kg SH, respectively. The ALI model was created after 6 h, and the rats were injected once a day intraperitoneally with c25-140 and SH (10, 20, and 40 mg/kg) for 72 h. On the eighth day, the rats were sacrificed to obtain samples after the rats were deeply anesthetized by an intraperitoneal injection of pentobarbital sodium (50 mg/kg).

### Establishment of ALI in the rats

According to previous literature [25], an ALI model was established by intratracheal instillation of LPS (5 mg/kg) using a 16G intravenous indwelling needle. The rats showed rapid breathing, and pathological examination showed signs of acute lung injury, indicating successful modeling. After LPS treatment for 24 h, 48 h, and 72 h, the mice were euthanized using sodium phenobarbital (50 mg/kg) and exsanguinated by cardiac puncture, and the lung tissue was removed for subsequent analysis.

### Wet-to-dry (W/D) lung weight ratio

To determine the wet weight of the rats, they were sacrificed after the rats were deeply anesthetized by an intraperitoneal injection of pentobarbital sodium (40 mg/kg), and their lungs were excised and weighed before being dried in an oven at 80 °C for 12 h to measure their dry weight. Then, to evaluate the pulmonary edema, we computed the W/D lung weight ratio.

### Real-time quantitative reverse transcription PCR (qRT-PCR)

Weigh about 0.6 g of each group of tissues was weighed using a homogenizer to break them up, add 1 mL of TRIzol RNA extraction reagent (Beyotime, Beijing, China), extract total RNA, and determine the purity (A260/A280 = 1.8 ~ 2.0), and the concentration of the samples was then reverse transcribed into cDNA. cDNA was obtained in accordance with Bestar SybrGreen qPCR Master Mix 10 uL, 0.8 uL of upstream primer, 0.8 uL of downstream primer, 2 uL of cDNA, 0.4 uL of ROX, and 6.0 µL of sterilized distilled water were used to form a 20 µL PCR amplification system. The CDS of each gene was queried in NCBI, and primers were designed by applying version 6.0 of the Premier software. The primer sequences used are listed in Table 1. The parameters for amplification entailed: pre-denaturation and denaturation processes at 95 °C for 2 min and 95 °C for 10 s, correspondingly. This was accompanied by annealing at 57 °C for 30 s, extension at 72 °C for 30 s, and 40 cycles. GAPDH was used as an internal reference, and the relative expression of target genes was analyzed using the 2^^−ΔΔCt^ method.

**Table 1:** Primers involved in real time PCR

Gene	The primer sequence (5’- 3’)
GPX4	F: GCCGAGTGTGGTTTACGAAT R: TTCCATTTGATGGCATTTCC
NCOA4	F: TCCAGAACCATCAGGACACA R: CTAGAAGGGCAAAGCCACAG
NOX1	F: GTGGCTTTGGTTCTCATGGT R: TGAGGACTCCTGCAACTCCT
FTH1	F: ATGATGTGGCCCTGAAGAAC R: GTGCACACTCCATTGCATTC
TRAF6	F: TCCCTGACGGTAAAGTGTCC R: TGTCTCCTGGGACAATCCTC
c-Myc	F: AACTTACAATCTGCGAGCCA R: AGCAGCTCGAATTTCTTCCAGATA
GAPDH	F: CAAACTGGTCTCCGAGAAGC R: GAATCGGACGAGGTACAGGA

### Hematoxylin-eosin (HE) staining

The lung tissue from the left lobe was fixed with 4% paraformaldehyde. When 48 h lapsed, the tissues were dehydrated, rendered transparent, and embedded in paraffin. HE staining was executed for staining the 4 μm lung sections for pathological changes under an optical microscope. The lung tissue damage (capillary congestion, fibrin exudation, cellular infiltration, and detachment of the bronchial epithelial cell) was scored with reference to the method of Mei et al. [26].

### Giemsa staining

The rats in each group were treated according to the group for 7 days. An intratracheal cannula containing three successive 1 mL aliquots of 0.9% sterile saline was essential in the collection of the bronchoalveolar lavage fluid (BALF) and the samples were centrifuged at 1500 rpm for 10 min at 4℃. The resuspension of cell pelletsin 0.9% sterile saline facilitated differential cell counts, which involved detecting alveolar epithelial cells, neutrophils, and eosinophils, using Giemsa staining.

### Immunohistochemical staining

Dewaxed paraffin sections were hydrated and incubated together with 3% hydrogen peroxide. They were afterward incubated utilizing anti-TRAF6 antibody (Beyotime, Shanghai, China) at 4 °C overnight. After washing, tissues were incubated with biotinylated sheep anti-rabbit IgG for 45 min at 37 °C. After washing, the sections were incubated with streptavidin-biotin complex (SABC) (Boster, Wuhan, China) for 30 min at 37 °C. After washing, diaminobenzidine (DAB, ZSGB–BIO, Beijing, China), was used to develop the sections and then stained with hematoxylin. This was then accompanied by dehydration, clearing, sealing, and observing the sections under a light microscope (Olympus BX41, Shanghai, China).

### Immunofluorescence

The right lung was removed for routine paraffin sectioning and dewaxing to water. Tissue sections were lysed in 0.05% Triton X-100 for 20 min, rinsed 3–5 times with phosphate-buffered saline (PBS), and 1% bovine serum was utilized to block them for 20 min. The Rabbit anti-c-Myc monoclonal antibody (Beyotime, Shanghai, China) was incubated for an entire night at 4 °C. We washed with PBS 3–5 times and added goat anti-rabbit fluorescent secondary antibody for 1 h at 20 °C. The secondary antibody was removed in a dark room and the tissue sections were washed 3–5 times by applying PBS. A 4′,6-diamidino-2-phenylindole (DAPI) stained the nucleibefore visualization under a Zeiss fluorescence microscope (Nanjing, China).

### Cell culture and grouping

The Shanghai Sixin Biotechnology (Shanghai, China) supplied rat type II alveolar epithelial cells (RLE-6TN). The cells were cultured in Dulbecco’s modified Eagle Medium (DMEM) culture medium enriched with 10% FBS at 37 °C in a 5% CO_2_ incubator. Logarithmic growth phase rat ATII cells were derived, digested, centrifuged, and resuspended in PBS. The cells were then inoculated onto a culture plate by adjusting the cell density. Corresponding stimulation and drug interference according to grouping were provided upon the cells binding to the wall. The specific groups and interventions were as follows: (1) Control (NC) group: rat ATII cells were cultured by adding cell culture solution Dulbecco’s PBS; (2) LPS group: rat ATII cells were incubated together with 100 ng/mL LPS; (3) RAS-selective lethal 3 (RSL3) group: rat ATII cells were incubated in conjunction with 35 mg/mL RSL3; (4) LPS + SH group: rat ATII cells were treated utilizing 10 µg/mL SH and 100 ng/mL LPS; (5) LPS + SH + (TRAF6-overexpression) OE-TRAF6 group: TRAF6-overexpressing rat ATII cells were treated utilizing 10 µg/mL SH and 100 ng/mL LPS; and (6) LPS + SH + knock-down (KD)-c-Myc group: KD-c-Myc rat ATII cells were treated utilizing 10 µg/mL SH and 100 ng/mL LPS.

Transfection experiments were undertaken according to Lipofectamine 2000 (Invitrogen) transfection instructions in the above experimental groups. TRAF6 overexpression treatments were performed by transfecting the pcDNA3.1 recombinant vector overexpressing TRAF6 and setting up a control null plasmid group. Knockdown of c-Myc was executed via adenoviral vectors carrying c-Myc short hairpin RNAs (Ad-sh-c-Myc-GFP, sh-c-Myc), which were derived by Hanbio (Shanghai, China), scrambled shRNA-GFP (shRNA) was utilized as the control. Three replicate wells were set up for each transfection experiment, and the efficiency of transfection was detected by qRT-PCR 48 h upon the completion of transfection.

### Transmission electron microscopy

An electron microscope fixative solution was utilized to fix the cells and stored at 4 °C as they awaited processing. Dehydrated and epoxy-immersed fixed cells were subjected to 48 h incubation at 60 °C after standing for 1 h. Subsequently, uranylacetate and lead citrate were utilized to stain ultrathin slices. Photographs were acquired via a transmission electron microscope (TEM, Spirit 120 kV, China).

### Cell counting kit-8 (CCK-8) assay

The CCK-8 kit was supplied by Beyotime (Shanghai, China). Rat ATII cells were collected using 0.25% trypsin and plated in 96-well plates at a rate of 5000 cells for each well, with three replicate wells per group. After the cells were attached to the wall, they were assigned into blank control, normal control, and experimental groups. After the medium was replaced, 10 µL of CCK-8 reagent was introduced into each well and the OD value was evaluated 2 h later at a wavelength of 450 nm utilizing a microplate reader.

### Ferrous iron colorimetric assay kit

Wuhan Elite Biotechnology Co., Ltd. (Wuhan, China) provided aferrous iron colorimetric assay kit. Rat lung tissues and ATII cells were collected to determine iron concentration. The assay was performed as per the manufacturer’s specifications.

### Enzyme-linked immunosorbent assay

In animal experiments, bronchoalveolar lavage fluid (BALF) was collected for 6 h following LPS exposure. The pro-inflammatory factors IL-6, TNF-α, IL-1β, and IL-18in BALF were measured using rat IL-6, IL-1β, TNF-a, andIL-18ELISA kits (Beyotime, Beijing, China). In cell experiments, the gathered cells were rinsed twice with PBS, centrifuged at 600 g for 10 min, resuspended in 1mL of reagent, and repeatedly freeze-thawed three times. We centrifuged the homogenate at 8000×*g* for 10 min at 4 °C using a centrifuge and collected the supernatant for assay. The supernatant was analyzed using rat GSH and rat MDA ELISA kits (Beijing Solaibao Technology Co., Ltd. Beijing, China).

### Determination of blood alkaline phosphatase (ALP), aspartate aminotransferase (AST), and lactate dehydrogenase (LDH)

The activities of ALP, AST, and LDH were measured using Olympus automatic biochemical analyzer (AU680, Tokyo, Japan). The reagent kit is provided by Shanghai Fosun Long March Medical Science Co., Ltd. (Shanghai, China). The determination method is carried out according to the instructions of the reagent kit.

### Determination of ROS activity

The Nanjing Jiancheng Bioengineering Institute (Nanjing, China) provided the ROS assay kit. A humidified incubator was utilized for culturing the cells at 37 °C. After the cells adhered to the wall, they were classified into a negative control group (medium only), a positive control group (active oxygen positive control solution), and a drug treatment group. The substitution of the medium with a fresh medium was executed, and this medium was left to stand for 24 h. Next, 200 µl DCFH-DA probe solution (working solution) was introduced into each well and incubated for 1 h. Next, the culture medium was removed, the cells were rinsed twice using PBS, the cell suspension was transferred into a centrifuge tube at 1000 rpm, and the supernatant was discarded following a 5-minute centrifugation. After aspirating the supernatant, cells were resuspended in PBS. Photographs were taken and scrutinized utilizing a fluorescence microscope and ImageJ software, correspondingly.

### Western blot (wb)

Rat ATII cells were lysed in radio immunoprecipitation assay lysis buffer (Beyotime). A total protein extraction kit (KeyGen Biotech, Nanjing, China) was used to extract total protein from the rat ATII cells. A bicinchoninic acid (BCA) kit (Cwbiotech, China) on the other hand determined the concentration of proteins. After denaturation, 30 µg of protein samples were loaded onto a 10% SDS-PAGE gel and transferred to a polyvinylidene fluoride membrane (Millipore, Bedford, USA), whereby, it was blocked for 2 h at room temperature with 5% skim milk and incubated at 4 °C over-night with the primary antibodies anti-FTH1 (ab266581, Abcam, USA), anti-GPX4 (AF7020, Beyotime, China), anti-NCOA4 (ab62495, Abcam, USA), andanti-NOX1 (ab131088, Abcam, USA). The cells were washed with PBS thrice, 5 min each time, and we added rabbit secondary antibody and incubated the cells at 37 °C for 4 h. Next, after rinsing thrice with PBST, an ECL luminescence reagent for imaging. The National Institutes of Health provided ImageJ software (version 1.8.0) that had a substantial function in the quantification of the relative expression of each protein.

### Statistical analysis

SPSS 26.0 (SPSS, Chicago, IL, USA) software executed all statistical analysis, and the findings were expressed as mean ± standard deviation. Images were drawn utilizing the GraphPad Prism 9 software (GraphPad, CA, USA). The experiments were triplicated independently. The t-test and the one-way ANOVA functioned in performing comparisons between two groups and comparisons across multiple groups, correspondingly. *P* < 0.05 was used to determine statistical significance.

## Results

### SH significantly ameliorated LPS-induced ALI in vivo

**Fig. 1:**
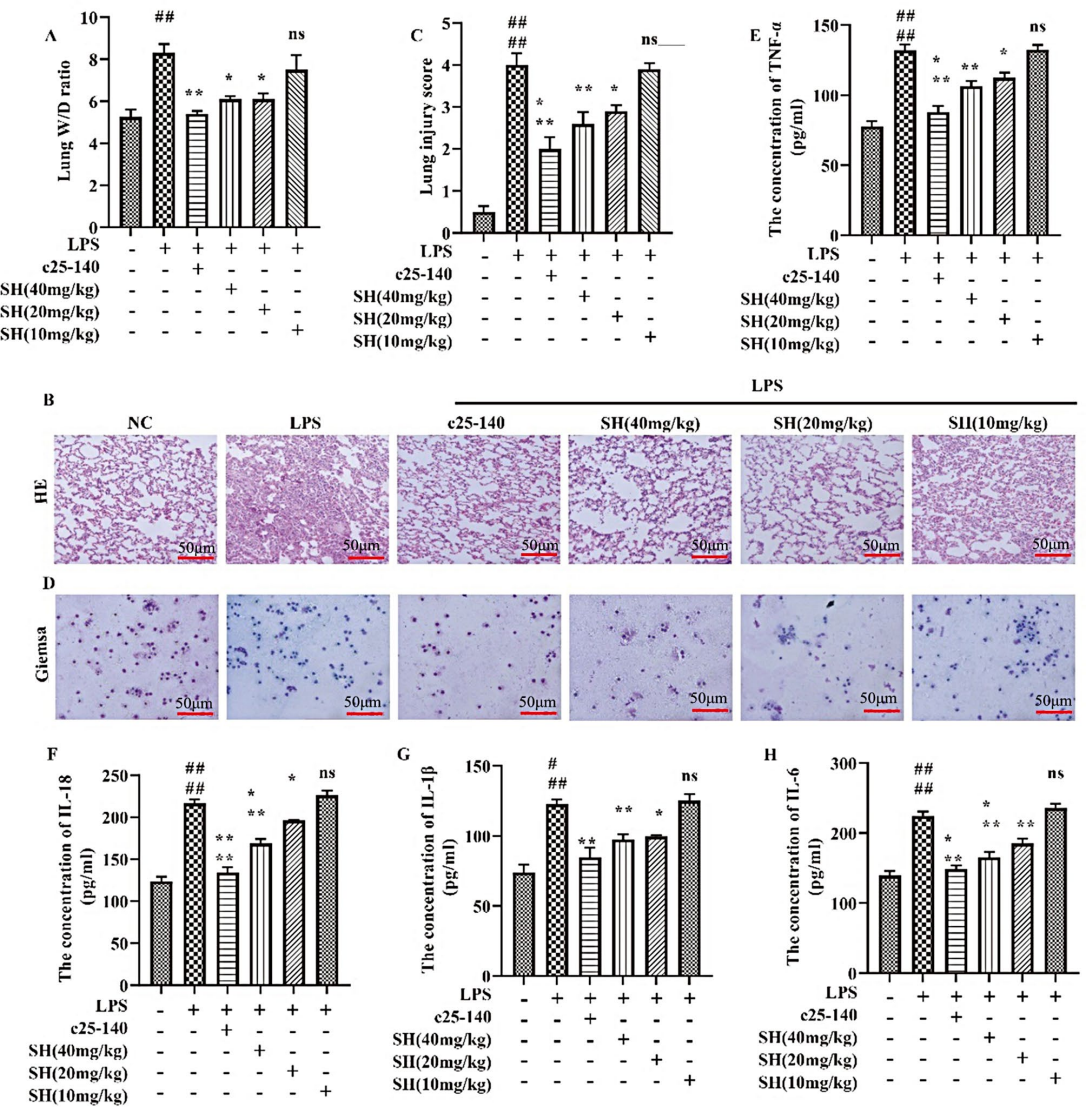
SH significantly ameliorated LPS-induced ALI in vivo. **(A)** The W/D ratio of lung tissues. **(B)** Representative bright field images of HE staining (scale bar = 50 μm). **(C)** Lung injury score. **(D)** Representative bright field images of Giemsa staining (scale bar = 50 μm), Neutrophils (blue), and eosinophils (purple). **(E**-**H)** ELISA detected the IL-18, IL-1β, and IL-6 concentrations in the BALF (*n* = 4 for every group). Data are shown as the mean ± SD, ^##^*p* < 0.01; ^###^*p* < 0.001; ^####^*p* < 0.0001; *p < 0.05; **p < 0.01; ***p < 0.001; ****p < 0.0001

In this study, rats were induced by the instillation of an intratracheal LPS to develop a rat model of ALI and ascertain the protective impact of SH towards LPS-induced ALI in rats. Figure 1A depicts the W/D ratio of lung tissues in the LPS group, which was affirmed to be higher than that in the control group and was substantially reduced upon c25-140 and SH (20 and 40 mg/kg) treatment. At the same time, in the LPS group, HE staining showed that the lung structure was severely damaged, with alveolar wall thickening, partial rupture of the alveolar septum, alveolar structure deformation, and epithelial cell degeneration, in contrast to the control group, and the rat lung damage was dramatically alleviated after c25-140 and SH treatment (Fig. 1B). Consistent with the histomorphological changes, the lung injury score increased significantly after LPS administration and was markedly reduced after c25-140 and SH treatment (Fig. 1C). Furthermore, Giemsa staining implied that c25-140 and SH treatment remarkedly alleviated the LPS-induced increment in neutrophil and eosinophil numbers in BALF (Fig. 1D). In the ELISA assay, c25-140 and SH treatment also reduced the LPS-induced surge in TNF-α, IL-18, IL-1β, and IL-6 in the BALF (Fig. 1E-H). These findings infer that SH significantly ameliorates LPS-induced ALI in vivo by alleviating inflammation, and inhibition of TRAF6 may ameliorate inflammatory responses and lung injury.

### SH significantly alleviated LPS-induced ferroptosis by inhibiting the TRAF6-c-Myc signaling pathway in vivo

**Fig. 2:**
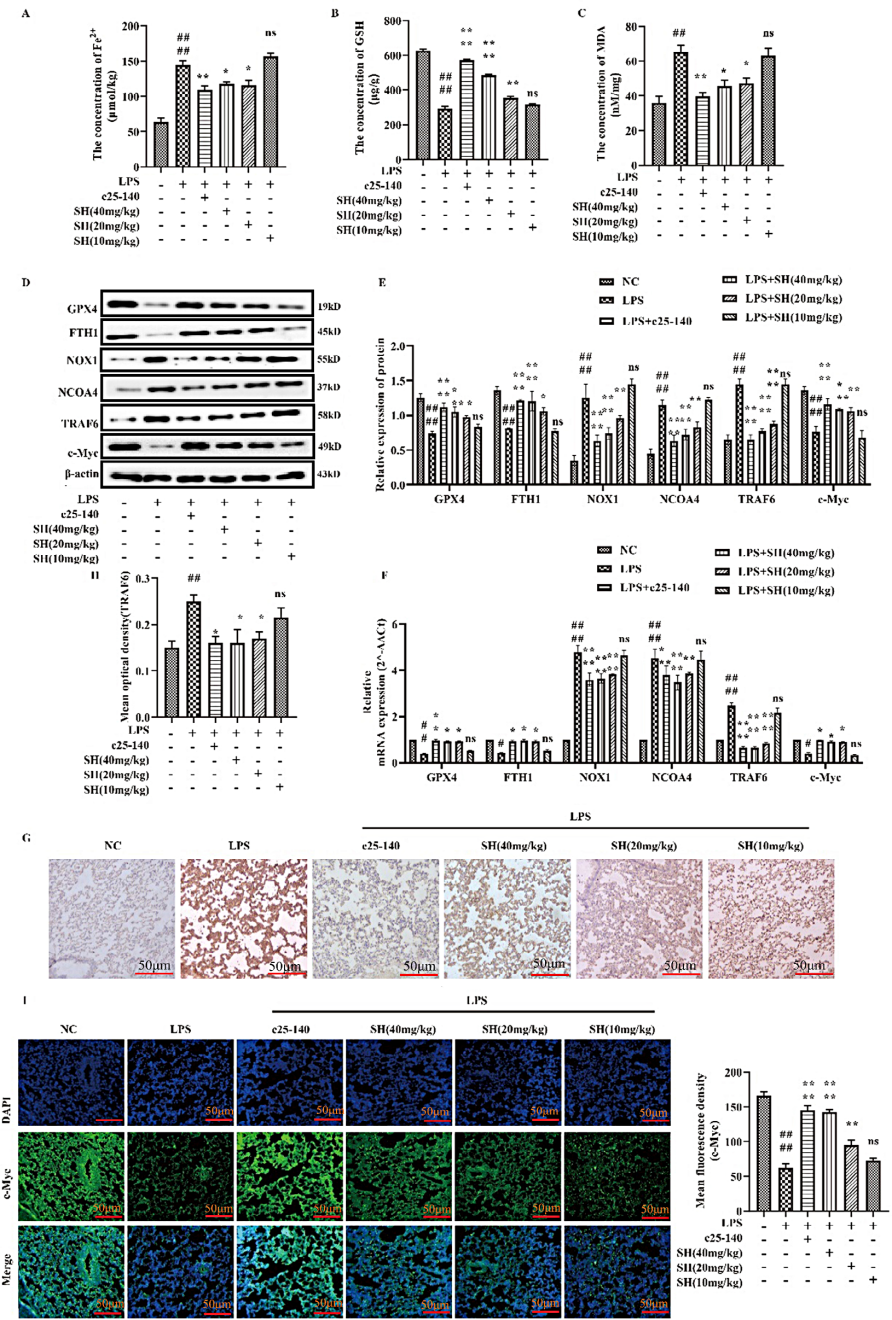
SH significantly attenuated LPS-induced ferroptosis by inhibiting the TRAF6-c-Myc signaling pathway in vivo. **(A)** The level of Fe^2+^ was evaluated by a ferrous iron colorimetric assay kit. **(B**-**C)** The levels of GSH and MDA was detected by Elisa kits. **(D**-**E)** Western blotting was used to detect the expression of GPX4, FTH1, NOX1, NCOA4, TRAF6, and c-Myc protein. **(F)** The mRNA expression of GPX4, FTH1, NOX1, NCOA4, TRAF6, and c-Myc was detected by qRT-PCR. **(G)** Representative immunohistochemical images of the expression of the TRAF6 in the lung tissue (scar bar = 50 μm). **(H)** The statistics of immunohistochemical staining. **(I)** Detection of c-Myc protein expression level by immunofluorescence staining (scar bar = 50 μm, *n* = 4 for every group). Data are shown as the mean ± SD, ^#^*p* < 0.05; ^##^*p* < 0.01; ^###^*p* < 0.001; ^####^*p* < 0.0001; *p < 0.05; **p < 0.01; ***p < 0.001; ****p < 0.0001

Ferroptosisis involved in LPS-induced ALI. When cells undergo ferroptosis, iron-dependent pooling of lipid peroxides causes mitochondrial contraction as well as membrane rupture, which in turn leads to cell death. As shown in Fig. 2A-C, the levels of Fe^2+^ and MDA were substantially heightened, and the level of GSH was diminished in lung tissues 6 h after LPS intra-tracheal instillation, which was significantly reversed after c25-140 and SH treatment. In addition, the protein expressions and genes linked to ferroptosis, such as GPX4, FTH1, NOX1, and NCOA4, were discerned utilizing western blotting and qRT-PCR. The expression levels of GPX4 and FTH1 were markedly diminished in the LPS group, whereas they were increased by c25-140 and SH treatment. In contrast, the expression of NCOA4 in conjunction with NOX1 was substantially heightened in the LPS group, while it was remarkably decreased by c25-140 and SH treatment (Fig. 2D-F). Finally, we delved into the functions of SH in LPS-induced ferroptosis in ALI. Immunohistochemical staining (Fig. 2G) and immunohistochemical staining (Fig. 2H) found that TRAF6 expression in LPS induced rats is significantly decreased after c25-140 and SH treatment compared to that in the LPS group. Immunofluorescence results showed that c25-140 and SH administration substantially alleviated the LPS-induced decrease in the fluorescence intensity of c-Myc in lung tissues (Fig. 2I). These findings imply that SH significantly inhibits LPS-induced ferroptosis by regulating the TRAF6-c-Myc signaling pathway in vivo.

### SH significantly attenuated LPS-induced ferroptosis in vitro

**Fig. 3:**
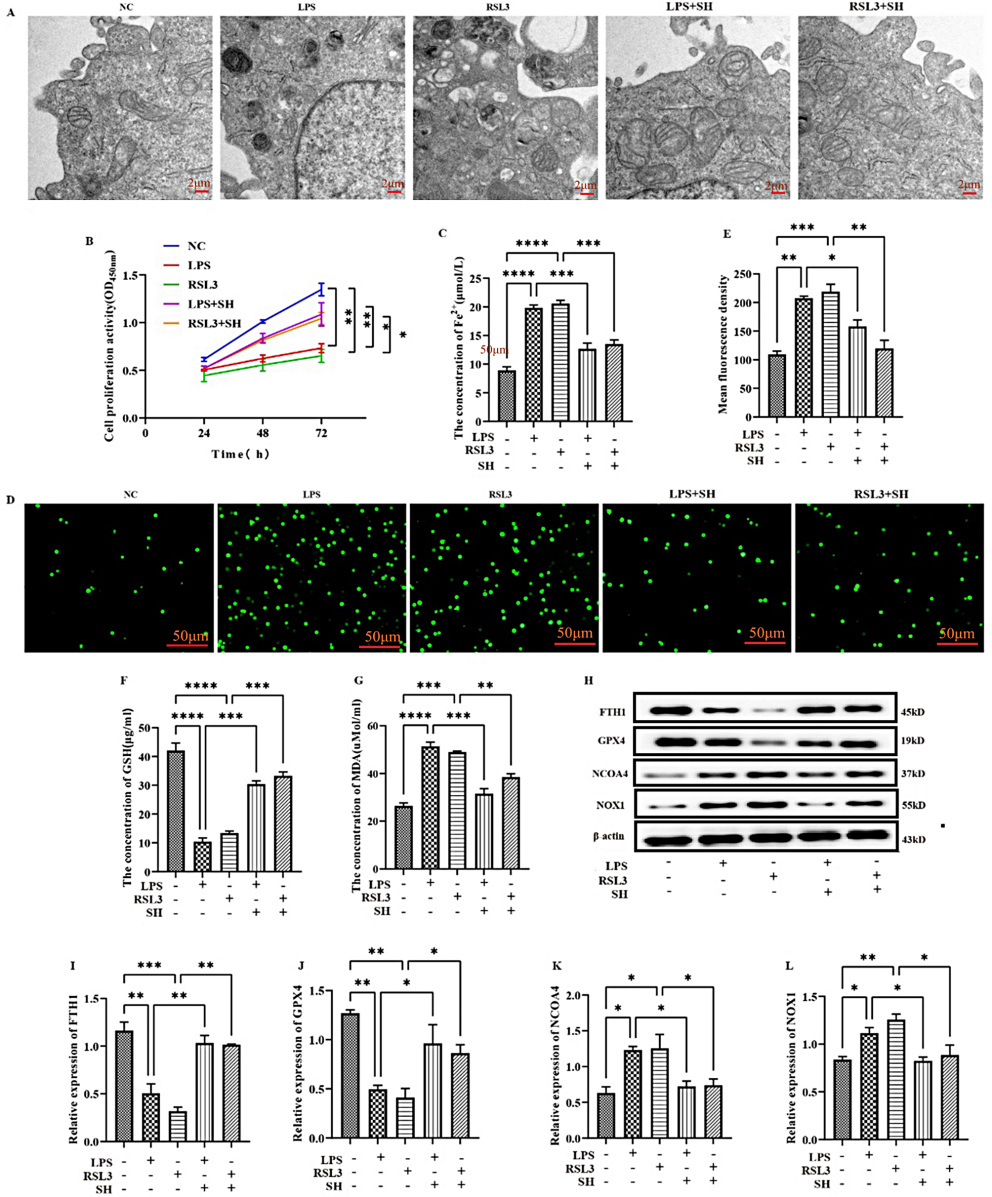
SH significantly attenuated LPS-induced ferroptosis in vitro. **(A)** Observation of mitochondrial structure by transmission electron microscopy in rat ATII cells (scale bar is 500 nm). **(B)** CCK8 was used for the detection of cell proliferation of rat ATII cells. **(C)** The cellular iron levels were evaluated by the ferrous iron colorimetric assay kit. **(D)** Representative ROS fluorescence picture of rat ATII cells. **(E)** ROS fluorescence intensity. **(F**-**G)** Concentrations of GSH and MDA. **(H**-**L)** Western blotting was used to detect the expression of FTH1, GPX4, NCOA4, and NOX1 protein. Data are shown as the mean ± SD, *p < 0.05; **p < 0.01; ***p < 0.001; ****p < 0.0001

To further illuminate the function of SH in LPS-induced ferroptosis, we came up with an LPS-mediated cell injury model on rat ATII cells in vitro. Transmission electron microscope images showed that a segment of the nuclear membrane was ruptured, nucleoli dissolved, mitochondriaenlarged, ruptured, and cristae vanished in the LPS and RSL3 group, while SH treatment effectively alleviated the destruction of mitochondrial structure (Fig. 3A). The CCK8 results indicated that SH treatment increased LPS-induced decrease in ATII cell viability (Fig. 3B). In addition, the level of intracellular ferrous ion (Fig. 3C), ROS fluorescence picture of rat ATII cells (Fig. 3D), and ROS fluorescence intensity (Fig. 3E) was increased after LPS and RSL3 stimulation while the above indicators were all reversed after treatment with SH. Finally, we uncovered the expression of ferroptosis-linked proteins. The expression of FTH1, GSH, and GPX4 was significantly decreased in the LPS and RSL3 groups, while they were potentiated by SH treatment. In contrast, the expression of MDA, NCOA4, and NOX1 was markedly heightened in LPS and RSL3 groups, while was remarkably decreased by SH treatment (Fig. 3F-L). In conclusion, these findings suggest that SH significantly attenuated LPS-induced ferroptosis in rat ATII cells.

#### SH significantly attenuated LPS-induced ferroptosis by inhibiting TRAF6-c-Myc signaling pathway in vitro

**Fig. 4:**
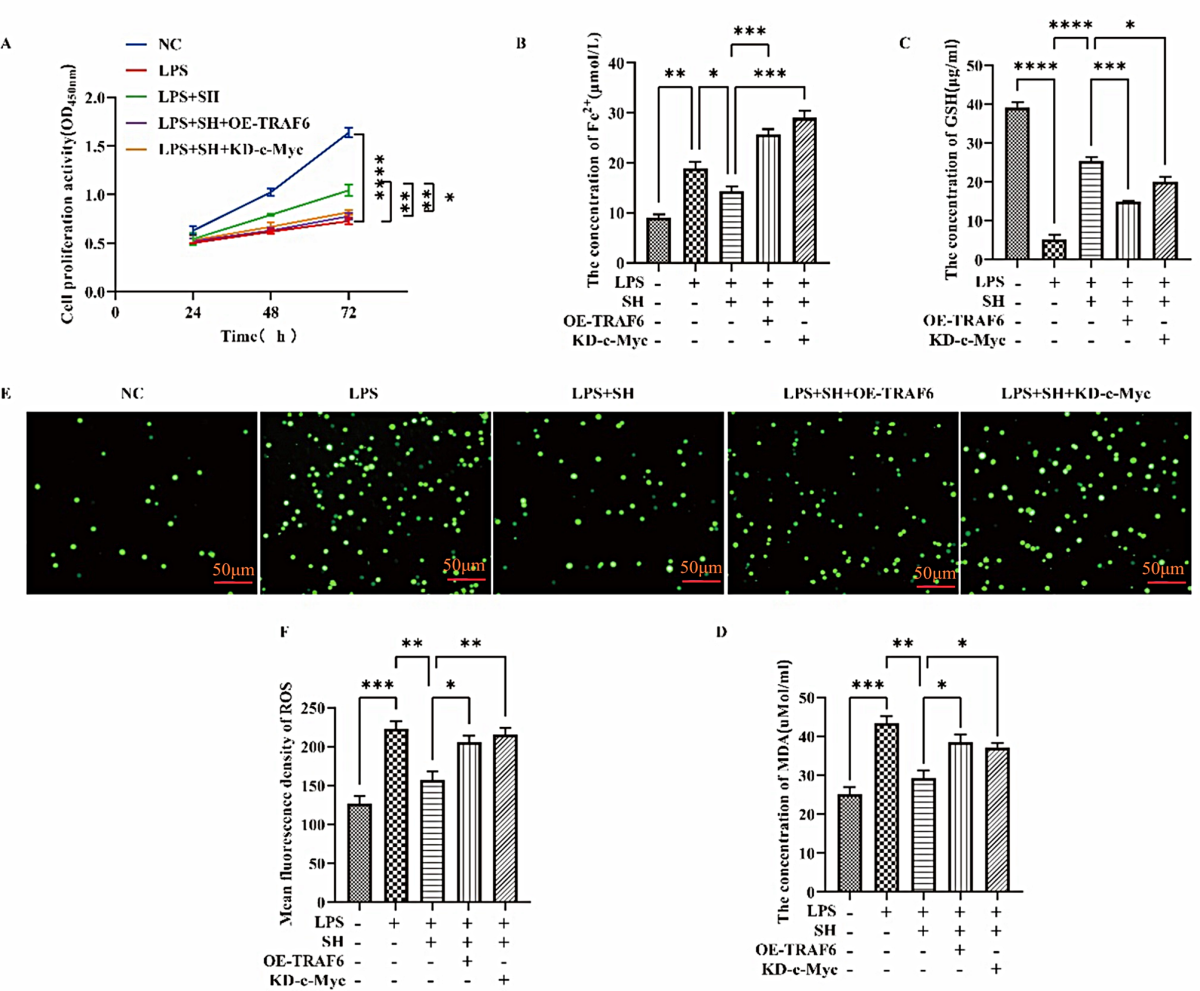
SH significantly attenuated LPS-induced ferroptosis by inhibiting the TRAF6-c-Myc signaling pathway in vitro. **(A)** CCK8 was used for the detection of cell proliferation of rat ATII cells. **(B)** The cellular iron levels were detected by the ferrous iron colorimetric assay kit. **(C**-**D)** Concentrations of GSH and MDA. **(E)** Representative ROS fluorescence picture of rat ATII cells. **(F)** Fluorescence intensity of ROS. Data are shown as the mean ± SD, *p < 0.05; **p < 0.01; ***p < 0.001; ****p < 0.0001

Furthermore, we delved into examining the in vitro function of SH on LPS-induced ferroptosis. Rat ATII cells transfected with TRAF6 or c-Myc overexpression constructs were incubated using two reagents, that is, 100 ng/mL and 10 µg/mL of LPS and SH for 24 h, correspondingly. Figure 4A manifests that cell viability was markedly reduced in the LPS group in contrast to the control group, while cell viability was significantly increased after SH treatment. Overexpression of TRAF6 and knockdown of c-Myc counteracted the advanced impact of SH on cell viability. In comparison to the control group, Fig. 4B-Faffirms that the concentration of intracellular iron ions, MDA content, and ROS levels was remarkably potentiated and GSH content on the other hand markedly lowered in the LPS group. However, the aforementioned indicators were markedly improved after SH treatment, whereas overexpression of TRAF6 and knockdown of c-Myc reversed the inhibitory influence of SH on ferroptosis. These findings affirm that SH significantly led to the attenuation of LPS-induced ferroptosis via modulation of the TRAF6-c-Myc signaling pathway in rat ATII cells.

### SH significantly improved liver injury in LPS-induced ALI in vivo

**Fig. 5:**
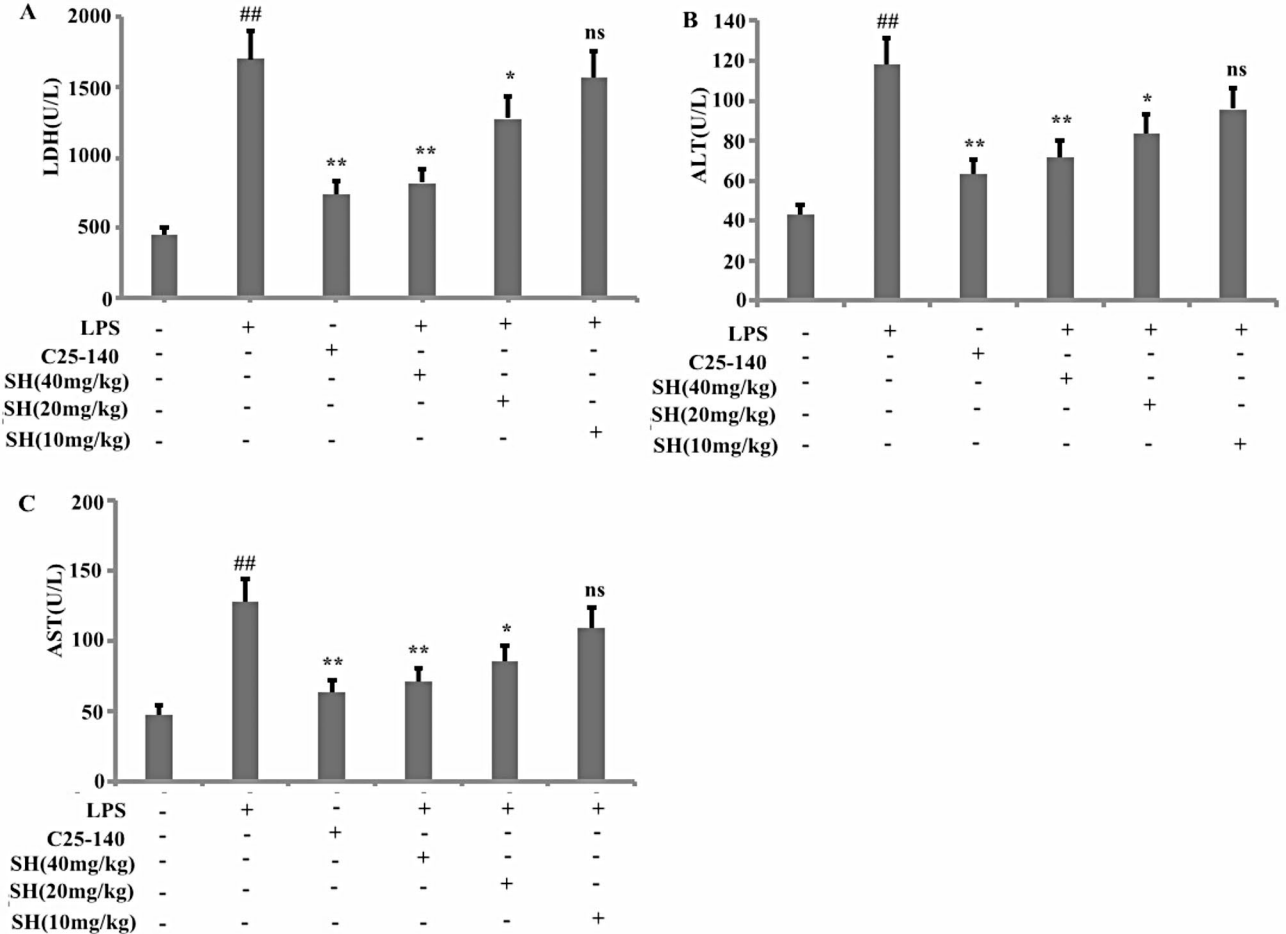
SH significantly improve liver injury in LPS induced ALI rats in vitro. The rats were induced by the instillation of an intratracheal LPS to develop a rat model of ALI and ascertain the protective impact of SH towards LPS-induced ALI in rats. The activities of ALP (**B**), AST (**C**), and LDH (**A**) were measured. Data are shown as the mean ± SD, ^#^*p* < 0.05; ^##^*p* < 0.01; ^###^*p* < 0.001; ^####^*p* < 0.0001; *p < 0.05; **p < 0.01; ***p < 0.001; ****p < 0.0001

In this study, rats were induced by the instillation of an intratracheal LPS to develop a rat model of ALI and ascertain the protective impact of SH towards liver injury in LPS-induced ALI rats. Figure 5A-C illustrate the serum AST, ALT, and LDH in the LPS group, which was affirmed to be higher than that in the control group and was substantially reduced upon c25-140 and SH (20 and 40 mg/kg) treatment. These findings infer that SH significantly ameliorates liver injury in LPS-induced ALI in vivo by alleviating inflammation, and inhibition of TRAF6 expression (Fig. 5A-C).

**Fig. 6:**
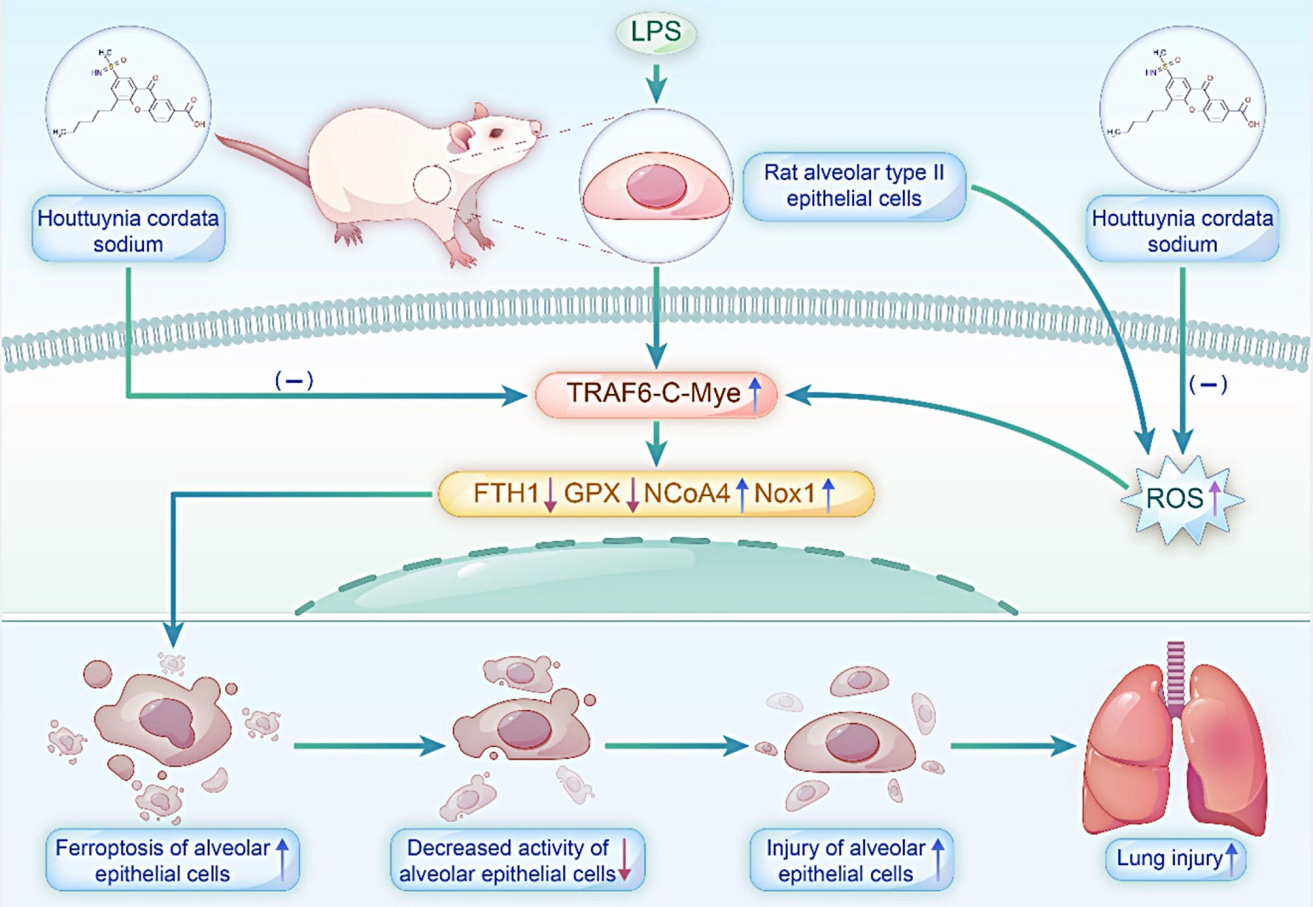
Mechanisms underlying the effects of SH on LPS-induced lung injury by inhibiting ferroptosis

## Discussion

ALI exhibits a complex pathogenesis that has not been fully elucidated. Unregulated inflammatory cytokine storms or inflammatory responses are important events throughout ALI, which can directly or indirectly result in the destruction of the microvascular endothelium as well as alveolar epithelium in the lungs [27]. Although substantial advancements have been realized in comprehending ALI pathogenesis. Its clinically effective therapeutic approaches are yet remarkably limited. ALI is mainly treated with a protective ventilation approach, utilization of hormones to support the treatment, and restrictive fluid management, but satisfactory results have not yet been achieved [28]. Therefore, the search for new drugs or therapies for ALI is of great value and significance.

SH has been extensively studied for its medicinal properties and has been shown to possess anti-inflammatory [17, 29] and antioxidative properties [30]. Emerging evidence has shown that SH ameliorates LPS-induced ALI by repressing the oxidative stress response and modulating the MAPK and NF-κB signaling pathways in vivo [17]. These findings align with the findings of this study. This study revealed that administration of SH successfully impeded LPS-induced pulmonary pathological alterations, increased inflammatory cells, and proinflammatory cytokines, and reverted the increment in the lung W/D ratio. The degree of these impacts was ascertained to be closely linked to the severity of ALI.

Xc-/GSH/GPX4, a classical antioxidant pathway, plays an important role inferroptosis inhibition. GSH has the potential to sustain cells from ferroptosis by conversion of toxic lipid peroxides into harmless fatty alcohols via the catalytic action of GPX4^33^. Therefore, GPX4 is considered to be a negative regulator of ferroptosis. Ferroptosis, driven by unrestricted peroxidation of lipids, is a dynamic process triggered by the invasion of ROS on lipids, especially PUFA in cell or organelle membranes [10], and results in morphological changes in the mitochondrial ultrastructure [32]. Pro-oxidant enzymes actively modulate this process, and it entails enzymes encompassing lipoxygenase (ALOX), cytochrome P450 oxidoreductase (POR), and NADPH oxidase (NOX) [33, 34]. Ferritinophagy functions in the control of ferroptosis. Ferritinophagy, a type of ferritin-related autophagy, initiates ferroptosis through ferritin degradation [35–37]. In ferritinophagy, the cargo receptor nuclear receptor coactivator 4 (NCOA4) selectively recognizes ferritin heavy polypeptide 1 (FTH1), causing the release of iron bound to FTH1, thereby increasing the labile iron pool (LIP), enhancing cellular ROS pooling, and inducing ferroptosis. When intracellular iron levels increase, FTH1 can rapidly bind and store ions to mitigate the cellular damage caused by increased iron levels [38, 39]. Consequently, we delved into the expression of ferroptosis-related proteins to determine whether ferroptosis occurred during LPS exposure. Interestingly, this study showed that SH administration effectively inhibited LPS-induced ferroptosis in vivo and in vitro. RSL3 is a potent ferroptosis-triggering agent that directly inhibits GPX4, which can promote ferroptosis. Therefore, in this study, we induced ferroptosis rat ATII cell models using RSL3.

Subsequently, we investigated the possible mechanisms underlying the involvement of SH in LPS-induced ALI. Tumor necrosis factor receptor-associated factor 6 (TRAF6), which holds one to seven zinc-finger and N-terminal ring-finger structural domains, belongs to the TRAF family that is distributed in the cytoplasm. TRAF6 mediates the tumor necrosis factor (TNF) receptor family and affects downstream signaling of the interleukin receptor/Toll-like receptor family [40]. In addition, TRAF6 binds to upstream signaling activators such as IL-1 and LPS, activating the NF-κB signaling pathway and promoting inflammation [41]. Prior literature has inferred that SH inhibits NF-κB activation in an LPS-induced ALI mouse model. Additionally, the NF-κB axis mediates ferroptosis in numerous diseases including liver cancer [42], glioblastoma [43], and cardiac dysfunction [44]. Furthermore, Li et al. showed that Sennoside A relieved inflammation, ferroptosis, and impairments in cognition in aging mice that have Alzheimer’s disease by decreasing TRAF6 [47]. Therefore, we explored whether the inhibitory impact of SH on the NF-κB signaling pathway is connected with its inhibitory effect on upstream TRAF6 signaling involved in ferroptosis. Interestingly, our study affirmed that SH could inhibit the TRAF6 signaling pathway participating in ferroptosis in vivo and in vitro.

The MYC family includes L-Myc, N-Myc, and c-Myc proteins. The oncogenic transcription factor c-Myc (also denoted as Myc) often regulates gene expression by directly attaching to the E-box elements situated at the promoter region of its target genes. Jin et al. showed that c-Myc can inhibit ferroptosis through NCOA4-mediated ferritin autophagy, thus reducing ROS and inhibiting mitophagy in ovarian cancer cells [46]. Pieces of literature have implied that overexpression of c-Myc can suppress erastin-induced ferroptosis among cancerous hepatocytes and additionally suggested that it inhibits ferroptosis [47]. Moreover, current research shows that pharmacological suppression of TRAF6 activity with the usage of TIP to modulate its pathway restrained c-Mycexpression as well as efficiently repressed hepatocellular carcinoma growth [48]. Starczynowski et al. demonstrated that loss of TRAF6 signaling bestows the development of acute myeloid leukemia in clonal hematopoiesis via MYCactivation [49]. Collectively, these studies showed that TRAF6 can target c-Myc-driven cancer cells. However, the role of TRAF6-c-Myc in ferroptosis has not yet been reported. Therefore, this study explored whether inhibition of ferroptosis by SH is related to TRAF6-c-Myc signaling. Surprisingly, the results of this study showed that SH can inhibit TRAF6 to activate the c-Myc signaling pathway, causing an inhibition of ferroptosis in vivo and in vitro.

We further investigated the effect of SH on liver injury in LPS induced ALI rats. Ours results found that LPS induced acute lung injury and also led to acute liver injury. Administering SH treatment improved LPS induced lung injury and also alleviated LPS induced liver injury.

### Limitations of the study

This study examined the protective mechanism of SH blocking TRAF6-c-Myc signaling onlipopolysaccharide-induced ALI. However, there are several limitations in this study. Firstly, the effect of TRAF6-c-Myc signaling on LPS induced ALI may not be limited to inflammation and ferroptosis -related protein pathways. Oxidative stress, copper induces cell death, autophagy, pyroptosis, apoptosis, and other proteinsand pathways might also be involved. Secondly, in addition to the TRAF6-c-Myc pathway, other pathways might also be associated with the protective effects of SH against LPS-induced ALI. The protective effect of SH might involve oxidative stress, apoptosis, pyroptosis, autophagy, and other processes. Further investigation must be conducted with in vivo and in vitro to determine the underlying mechanisms.

## Conclusion

In summary, SH attenuates ferroptosis by regulating the TRAF6-c-Myc signaling pathway in LPS-induced ALI as per our findings. This study elucidates the mode by which LPS controls ferroptosis-induced alveolar epithelial cell injury and provides a possible therapeutic target for the therapeutic management of ALI utilizing SH (Fig. 6).

## Electronic supplementary material

Below is the link to the electronic supplementary material.

Supplementary Material 1

## Data Availability

No datasets were generated or analysed during the current study.

## Abbreviations

SH: Sodium Houttuyniae
TRAF6: Tumor necrosis factor receptor-associated factor 6
NCOA4: Nuclear receptor coactivator 4
TNF: Tumor necrosis factor
LPS: Lipopolysaccharide
ALI: Acute lung injury
CCK-8: Cell Counting Kit-8
MTT: 3-(45)-dimethylthiahiazo(-z-y1)-35-di-phenytetrazoliumromide
IL-1β: Interleukin-1β
IL-18: Interleukin-18
IL-6: Interleukin-6
RT‒PCR: Reverse transcription polymerase chain reaction
ROS: Reactive oxygen species
NOX: NADPH oxidase
GPX4: Glutathione peroxidase 4
SD: Sprague Dawley
c-Myc: Myc gene
FTH1: Ferritin heavy polypeptide 1
ALOX: Lipoxygenase
LIP: Labile iron pool
SABC: Streptavidin-biotin complex
BALF: Bronchoalveolar lavage fluid
PBS: Phosphate-buffered saline
CCK-8: Cell counting kit-8
RLE-6TN: Rat type II alveolar epithelial cells
RSL3: RAS-selective lethal 3
