# Supplementary material for: Sodium Houttuyniae attenuates ferroptosis by regulating TRAF6-c-Myc signaling pathways in lipopolysaccharide-induced acute lung injury (ALI)
Source: BMC Pharmacol Toxicol. 2024 Sep 6;25:63. doi: 10.1186/s40360-024-00787-x (PMC11380410; doi:10.1186/s40360-024-00787-x)
Supplement: Supplementary file 1 — Supplementary Material 1 [file 40360_2024_787_MOESM1_ESM.docx]

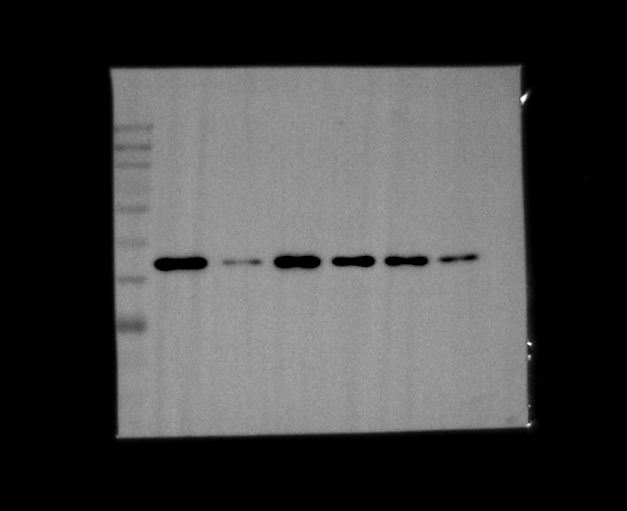


**Figure2 D: The protein expression of c-myc**

**
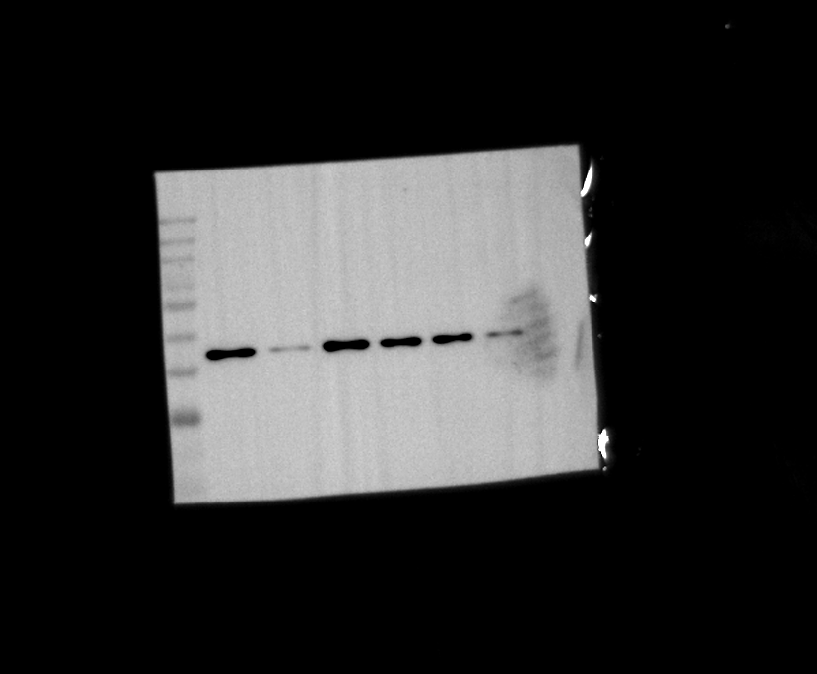
**

**Figure2 D: The protein expression of FTH1**

**
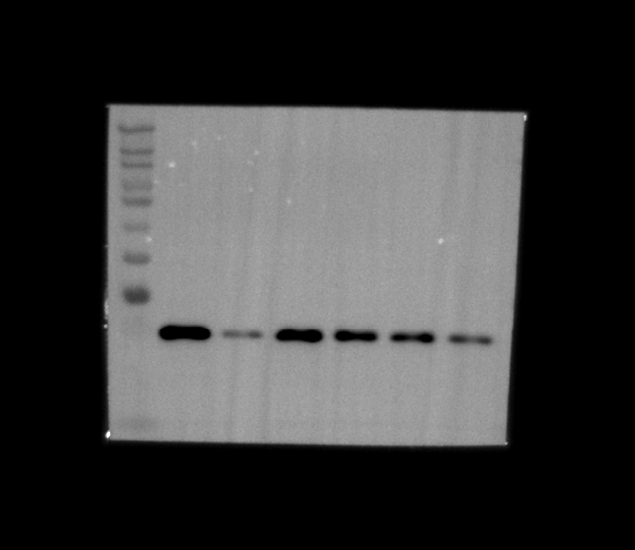
**

**Figure2 D: The protein expression of GPX4**

**
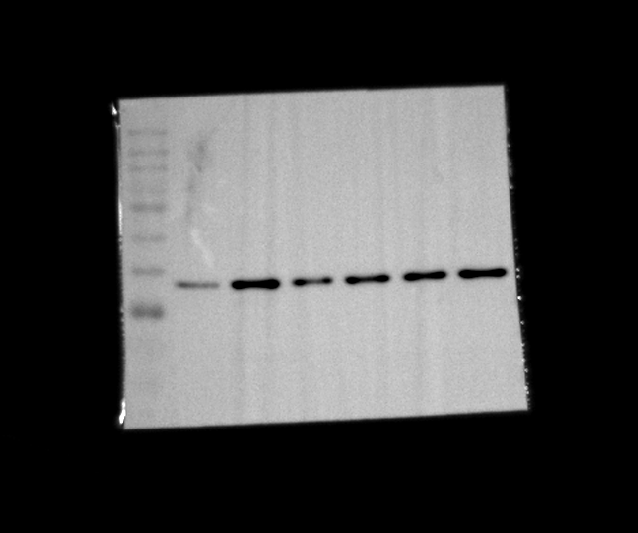
**

**Figure2 D: The protein expression of NCOA4**

**
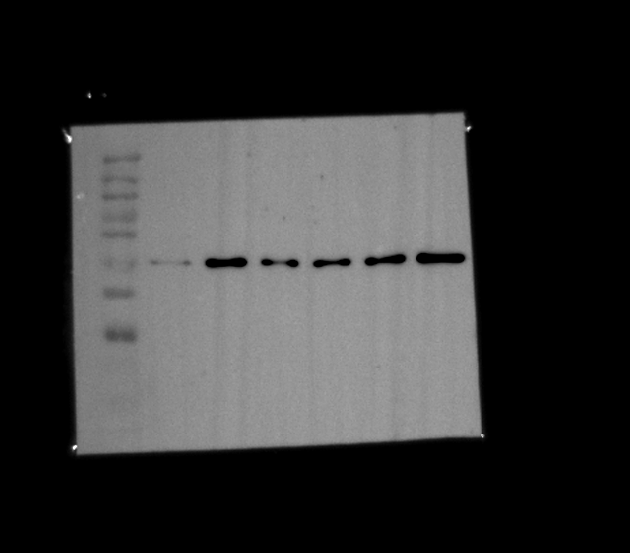
**

**Figure2 D: The protein expression of NOX1**

**
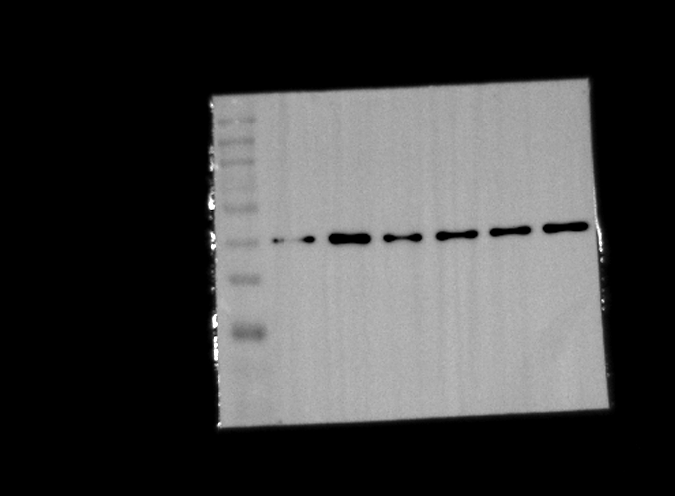
**

**Figure2 D: The protein expression of TRAF6**

**
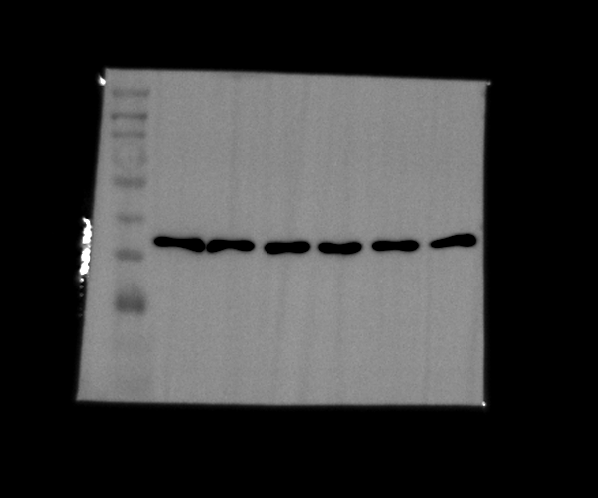
**

**Figure2 D:** **The protein expression ofβ-actin**

**
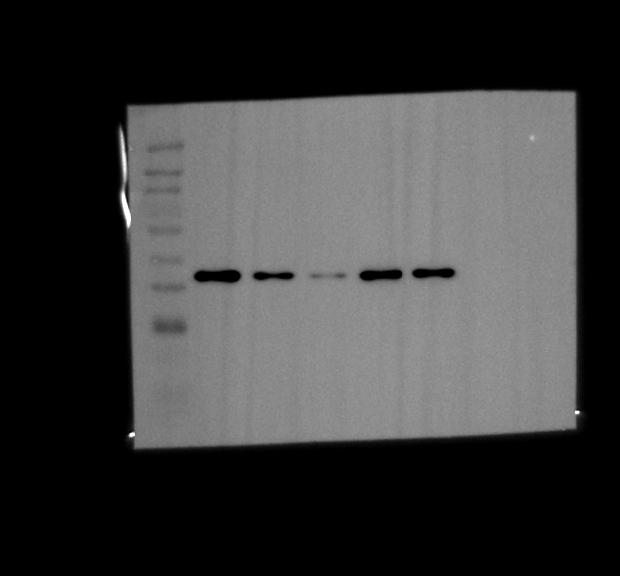
**

**Figure3H: The protein expression of FTH1**

**
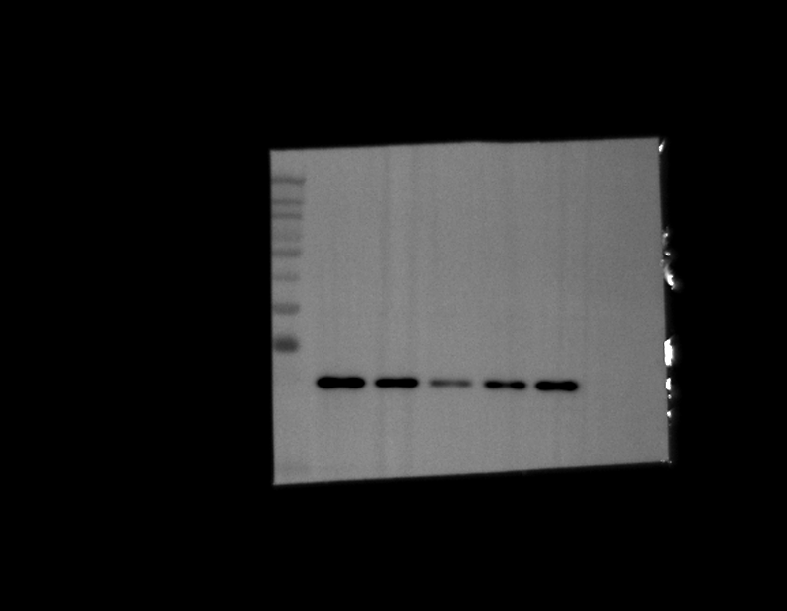
**

**Figure3H: The protein expression of GPX4**

**
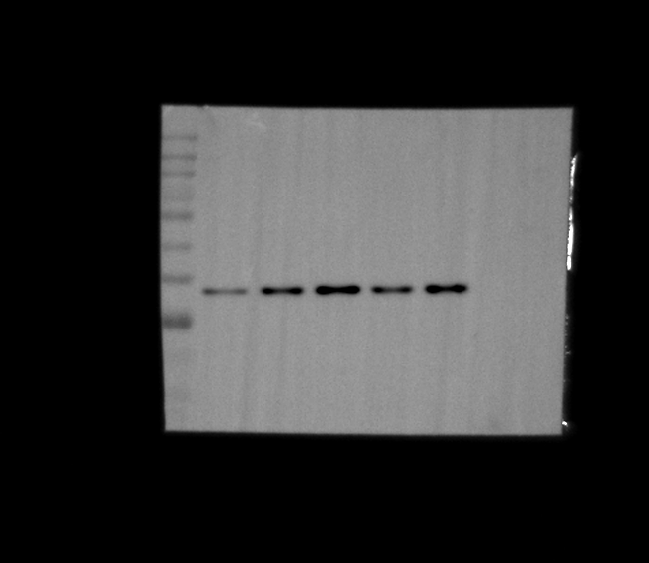
**

**Figure3H: The protein expression of NCOA4**

**
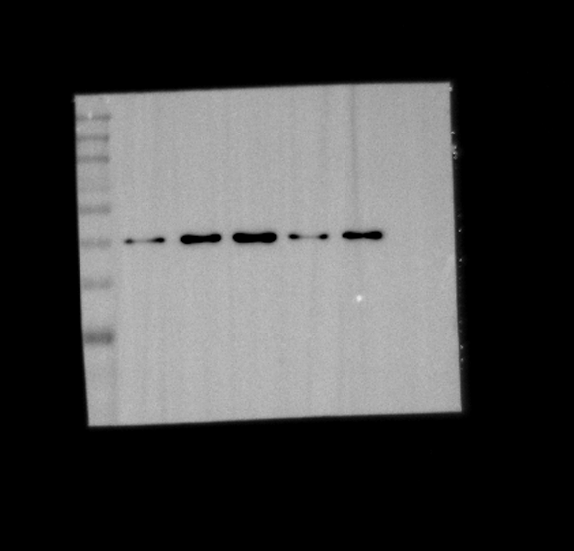
**

**Figure3H: The protein expression of NOX1**

**
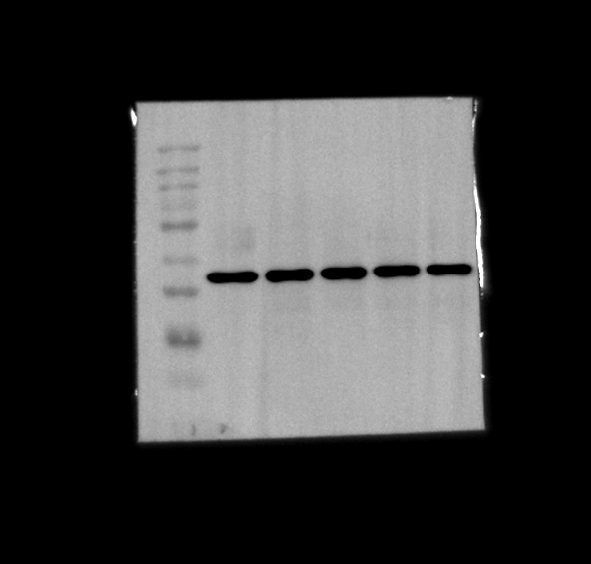
**

**Figure3H: The protein expression ofβ-actin**
